# Mechanical, Wear and Thermal Behavior of Polyethylene Blended with Graphite Treated in Ball Milling

**DOI:** 10.3390/polym13060975

**Published:** 2021-03-22

**Authors:** Annamaria Visco, Antonio Grasso, Giuseppe Recca, Domenico Carmelo Carbone, Alessandro Pistone

**Affiliations:** 1 Department of Engineering, University of Messina, Contrada Di Dio, I-98166 Messina, Italy; antonio.grasso@unime.it; 2 Institute for Polymers, Composites and Biomaterials-CNR IPCB, Via Paolo Gaifami 18, 95126 Catania, Italy; giuseppe.recca@cnr.it (G.R.); domenicocarmelo.carbone@cnr.it (D.C.C.)

**Keywords:** HDPE, ball-milled graphite, blends, tensile, torque, mechanical test, wear test

## Abstract

Additive manufacturing, civil, and biomechanical applications are among the most important sectors, where the filler’s presence can significantly improve the quality of polymeric products blends. The high market demand of new low-cost material to be used as shock absorbers and mechanical joints arouses our curiosity to study a relatively common commercial polymer and filler. The possible improvement by blending high-density polyethylene (HDPE) and graphite was investigated for these sectors. To achieve this objective, we have prepared HDPE/graphite nanocomposites following mechanical treatment to understand which parameter provides the researched properties. As widely reported in the literature, milling treatment leads to the decrease of the particle size and the exfoliation of graphitic layers. Therefore, graphite has been previously treated with a ball mill for different times (1–16 h) to enhance its lubricating action. We checked an improvement in stiffness, yielding strength, thermal stability, and, in particularly, wear resistance that increased by 65% with respect to that of polyethylene (PE). A treatment time of eight hours in ball milling could be enough to give an appreciable improvement. The wear behavior of HDPE with treated graphite has not been deeply investigated so far, and it could be important because HDPE is considered a “carrier polymer” for different low-friction applications.

## 1. Introduction

Polyethylene (PE) is a chemically inert material with a low coefficient of friction, which is flexible, ductile, mechanically resistant, tough, nontoxic and sterilizable with gas or disinfectants [1,2]. It is easily workable with the normal technologies of transformation of thermoplastic materials [3]. Among all the various types of PE, high-density polyethylene (HDPE) is white, translucent and very crystalline. Thanks to its remarkable properties of chemical inertia, HDPE is used to produce biomaterials (from containers for chemical or pharmaceutical substances, bags, drainage tubes, disposable gloves, catheters, etc.) that come into direct contact with both cells and tissues [4].

To further improve the physical and mechanical features of polymers, it is possible to add carbonaceous fillers such as graphite, carbon fibers, carbon nanotubes, and graphene [5,6,7]. It is known that carbon nanofibers and graphite, although they do not offer the same mechanical and physical improvement compared to nanotubes and graphene, significantly reduce high costs and complex processing associated with the production of carbon nanotubes and graphene [8]. Naturally unmodified graphite (NG) is an allotropic form of carbon organized in hexagonal planes made up of carbon atoms, each covalently bonded to three neighboring atoms. These planes are sheets that are only one atom thick and make up the layers of graphene [9,10]. Graphite is, hence, the accumulation of monatomic layers of graphene arranged with one on top of the other, joined by weak Van der Walls bonds. Through the chemical path, these bonds can be altered by inserting acidic components into the solution that catalyze each other and form a pressure difference such as breaking these surfaces (Hummer method) [11,12]. In this way, an exfoliated or expanded graphite (EG) layer can be produced. Similarly, stacks of nanosheets can be obtained by rapid heating of a Graphite Intercalation Compound (GIC) [13]. Exfoliated or EG is also called as graphite nanoplatelets (GNPs), graphite nanosheets (GNs) or graphite nanoflakes (GNFs), being a type of graphitic nanofillers composed of stacked two-dimensional (2D) graphene sheets and having an outstanding electrical, thermal, and mechanical properties [14].

In addition to chemical or thermal methods, the exfoliation of graphite can take place with a mechanical procedure, such as the micromechanical exfoliation of graphite. These procedures are based on the application of a force to the surface of oriented graphite crystals, so that the attractive Van der Waals force which binds one of graphene planes above the other is contrasted to unfold the crystalline layers until a single layer is obtained [11,15]. The “Scotch method” (Nobel Prize, 2004) tries to open these planes through an adhesive action from the contact between graphite with some materials with the consequent unfolding of the graphene along the contact surface between the two materials. This method demonstrates that, directly from graphite, it is possible to exfoliate graphene or to unfold it in planes with a monatomic thickness of carbon sp2 simply by applying the tearing action of one layer of a scotch tape on a graphite plane and exerting a breakout force [15].

Graphite is usually added in several different polymers, both thermosets (such as epoxy resin, and polyester resin) and thermoplastics (such as polystyrene, polymethyl methacrylate, ethylene vinyl acetate, polyurethane, and polyvinylidene fluoride). The common result is that the treated graphite positively changes the polymer’s mechanical and insulating features [8,9].

Some authors have specifically studied the effect of graphite fillers on the tensile mechanical and electrical features of HDPE-based composites [16,17,18,19,20,21]. They were usually obtained by melt blending. The authors employed different weight amounts of graphite fillers (type: NG, EG, or GNPs) [9,14]. Graphite fillers were of different sizes, different distributions of particle size, and different specific surface areas. They checked the changes in electrical, thermal, and tensile mechanical properties. The results suggested that these features have been generally improved compared to those of neat HDPE. It was also pointed out that the worsening of other properties (or the lower-than-expected result) could be attributed to the poor affinity between the polar graphite and the nonpolar matrix during the realization of the nanocomposite [17]. Thus, particle volume fractions, aspect ratio, number, and distribution of particles, type of the boundary between the filler and the polymeric matrix, and particle’s shape have great effects on the resulting nanocomposite properties [22,23,24].

In addition, Jiang and Drzal investigated the flexural features and the impact strength of HDPE reinforced with exfoliated GNPs [25]. The authors highlighted the importance of filler dispersion in the polymer matrix and the filler’s adhesion to the matrix in order to have improved flexural features. Graphene can be functionalized to obtain graphene oxide and to decrease the difference in the polarity of components in the polar matrix.

Graphite could be also considered to improve the wear resistance of polymers, since it is an additive capable of increasing tribological properties [26,27,28]. This occurs thanks to the known lubricating properties of carbonaceous layers [29].

Thus, in this paper we prepared a nanocomposite blend made by HDPE and ball milled graphite (obtained by mechanical treatment in a ball milling machine for different times and with reduced particle’s size). The nanocomposites were characterized by torque, tensile, wear, and calorimetric analysis. To the best of our knowledge, the improvement of the wear resistance property of HDPE with BMG has not been deeply investigated so far. Applications of low-friction HDPE are, for instance, to produce water pipelines with improved fluo-dynamic [30] or work as a “carrier-polymer” which is easily mixable with a specific filler and then dispersible in a higher-viscosity and “difficult-to be-processed” polymer, such as the ultrahigh-molecular-weight polyethylene (UHMWPE). Then, the blend—filled HDPE/UHMWPE results in a higher-wear-resistance PE for biomedical use [26,31,32,33] or for use in civil engineering. An example application in civil engineering is the pendulum isolator, a seismic isolation device used in buildings and bridges for the earthquake protection [34,35].

## 2. Materials and Methods

**Figure 1:**
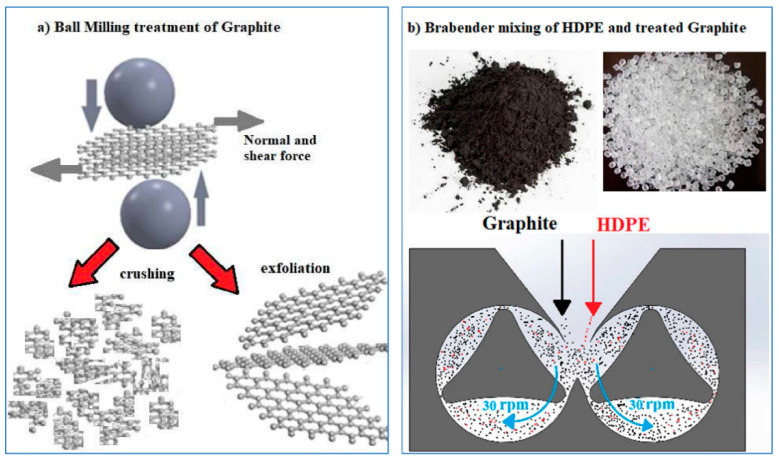
(**a**) A Solidworks^®^ representation of the graphite exfoliation/crushing by ball milling treatment. (**b**) The materials mixing in a Brabender Plasticorder.

Graphite powder (Sigma-Aldrich, Merck KGaA, Darmstadt, Germany); pureness: 99.9%; code: 282863) was previously ground by a ball mill (Retsch, model: v5001) at a frequency of 20 Hz for varied times (i.e., 1, 2, 4, 6, 8, and 16 h), to give shear and compression forces also useful to separate graphene layers from the graphite bulk and to crush the graphite powder in smaller size (Figure 1a). The ball mill was composed of two jars, of which the volume was 25 mL. The spherical stainless-steel ball (inserted in each jar) had a diameter of 15 mm and a volume of 14.1 mL. Furthermore, 1 g of graphite was inserted into each jar for its mechanical treatment. Ball milling treatments were set for a maximum consecutive time of 1 h, always followed by a rest period of 15 min before restarting the next treatment (Figure 1b). Graphite particle size was evaluated by dynamic light scattering (DLS) measurements using a Malvern Zetasizer Nano S instrument equipped with 4 mW He–Ne laser operating at a wavelength of 633 nm. Measurements were performed on samples dispersed in ethanol at 20 °C, with a detection angle of 90° and processed with Zetasizer software in z-average mode. Graphite’s morphology was observed by a scanning electron microscope (FEI Quanta FEG450 microscope). The scanning electron microscope was operated at an accelerating voltage of 15 kV and in low-vacuum mode. The samples adhered to aluminum holders by means of a graphitic adhesive. Images were taken at magnifications of 15 and 150 Kx.

Then, HDPE in pellets (supplied by Versalis; code: ERACLENE-MP90) and the graphite powder ball-milled at the different times were put inside the static mixer chamber of a Brabender Plasticorder (model PL2000) at 180 °C at a speed rate of 30 rpm and for a mixing time of 15 min. The ball-milled graphite amount was fixed at a 0.3 weight percentage in all the blends to have the best improvement in mechanical behavior, according to the experimental evidences of [36,37]. These authors highlighted in their studies that this filler amount was optimal to improve the polymer’s mechanical behavior (tensile strength, fracture strain, and yield strength). The obtained blends were codified as “PE-G” followed by a number, which represented the time in the ball milling treatment of graphite, as resumed in Table 1.

The torque value (expressed in Nm) of each material during the mixing time was recorded. The torque (that was the index of the viscosity variation of the blended materials in a molten state) was measured at predetermined time intervals (i.e., one minute) with a transducer interfaced with the specific Brabender software operated on a PC.

The materials obtained from the mixing process were placed in a stainless-steel mold. The materials with a length of 12 cm, a width of 12 cm, and a thickness of 1 mm were used for the tensile test, and the materials with a length of 2 cm, a width of 2 cm, and a thickness of 2 mm were employed for the wear test. Subsequently, they were compressed at 180 °C and 100 bar for 10 min by means of a hot press machine (DGTS srl, Verduggio, Monza Brianza, Italy), with Teflon release films (thickness: 300 μm) and subsequently water-cooled. Dog bone samples for the tensile test were obtained from a Ray-Ran cutter (according to the ASTM D-638 standard).

The static tensile tests of the pure HDPE and the one blended with ball-milled graphite at different times (from 0 to 16 h which was the highest ball milling time) were performed using a Lloyd LR10K universal machine. Specimens used had the type V geometry, according to the ASTM D638-10. The crosshead speed was 10 mm/min. Tensile mechanical parameters, obtained from the resulting stress–strain curves, were as following: Young’s modulus (E, MPa), yield stress (σ_y_, MPa), yield strain (ε_y_, %), stress at break (σ_b_, MPa), strain at break (ε_b_, %), work at rupture (W_b_, J), and maximum load (Load, N). Resulting values were the average of 10 samples for each test.

Wear resistance measurements were performed in a pin-on-disc wear tester in air and at room temperature by using a Ti-6Al-4V alloy with 2 mm in diameter. The pin-on-disc system gave a circular-shape wear trajectory with a testing load of 30 N, speeds of 0.25, 0.50, and 1.00 rad/s, and a test duration of 2500 turns for each measurement. For each sample, the specific wear rate *Wsp* (mm^3^/Nm) was calculated as:

(1) $$Wsp\;=\;\frac{\Delta m}{\rho \;L\;F_{n}},\;$$

where ∆m (mg) is the mass loss of the specimen, *ρ*(g/mL) is the density, *F_n_* (N) is the normal load, and *L* (m) is the total sliding distance. The final value of the specific wear rate *Wsp* was determined by the average of the *Wsp* values of n.3 polymeric samples (for each nanocomposite obtained) [29,30]. The mass loss and the density were evaluated by a high-sensitivity electronic weighing balance (Explorer pro, OHAUS Corporation, Parsippany, NJ-USA, EP 214C) with an accuracy of 10^4^ g. The density of pure PE was 0.9604 g/cm^3^, and that of the nanocomposite with 0.3 wt % of ball milled graphite was 0.965 g/cm^3^.

Thermal analyses were conducted by means of differential scanning calorimetry (DSC) using a TA Instruments DSC Q100, from room temperature to 200 °C, with a heating rate of 10 °C/min and water cooling. The crystalline degree of HDPE, *χ*, was calculated using the following equation:

(2) $$\chi =\;\frac{\Delta H_{m}}{\varphi \cdot \Delta H_{0}},$$

where

Δ*H_m_* (J/g) is the melting enthalpy, φ is the weight fraction of the studied material and
Δ*H*_0_ of 293 (J/g) is the theoretical enthalpy of fusion of a polymer crystal with infinite extension [38].

The TGA of the graphite samples was performed by an SDT Q600 thermogravimetric analyzer, in argon from 50 to 900 °C at a rate of 20 °C/min.

## 3. Results and Discussion

**Figure 2:**
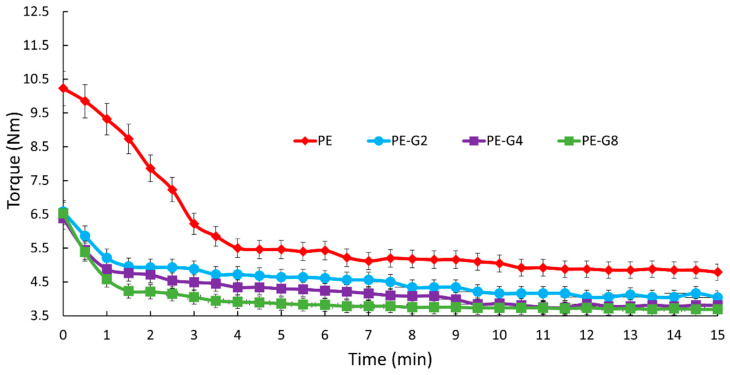
Torque vs. time for PE, PE-G2, PE-G4, and PE-G8 samples.

Figure 2 shows the trend of the torque as a function of the mixing time (in the Brabender mixer) of the pure HDPE and the ones with graphite ball-milled for different times. For the clarity of graphic presentation only those at a low time (2 h), an average time (4 h), and a high time (8 h) are shown. The initial torque value of the pure HDPE was about 10 Nm. It rapidly decreased during the first 5–6 min of mixing and then became constant around a value of 5 Nm. The addition of 0.3 wt % of ball-milled graphite lowered the torque value from the initial moment of all the samples (6.5 Nm). In the PE-G2 sample, the torque further decreased after a few minutes of mixing and then became constant around a value of 4 Nm (after 15 min).

The presence of graphite ball-milled for 8 h further lowered the torque value to 3.7 Nm (after 15 min of mixing). In general, the torque progressively decreased. The more the ball-milled graphite, the longer the graphite treatment time. The torques of these samples are shown in the order:

PE > PE-G0 > PE-G1 > PE-G2 > PE-G4 > PE-G6 > PE-G8 > PE-G16.

This indicated that the presence of ball-milled graphite decreased the friction of the HDPE inside the mixing chamber. The more the ball-milled graphite, the longer the ball milling time.

**Figure 3:**
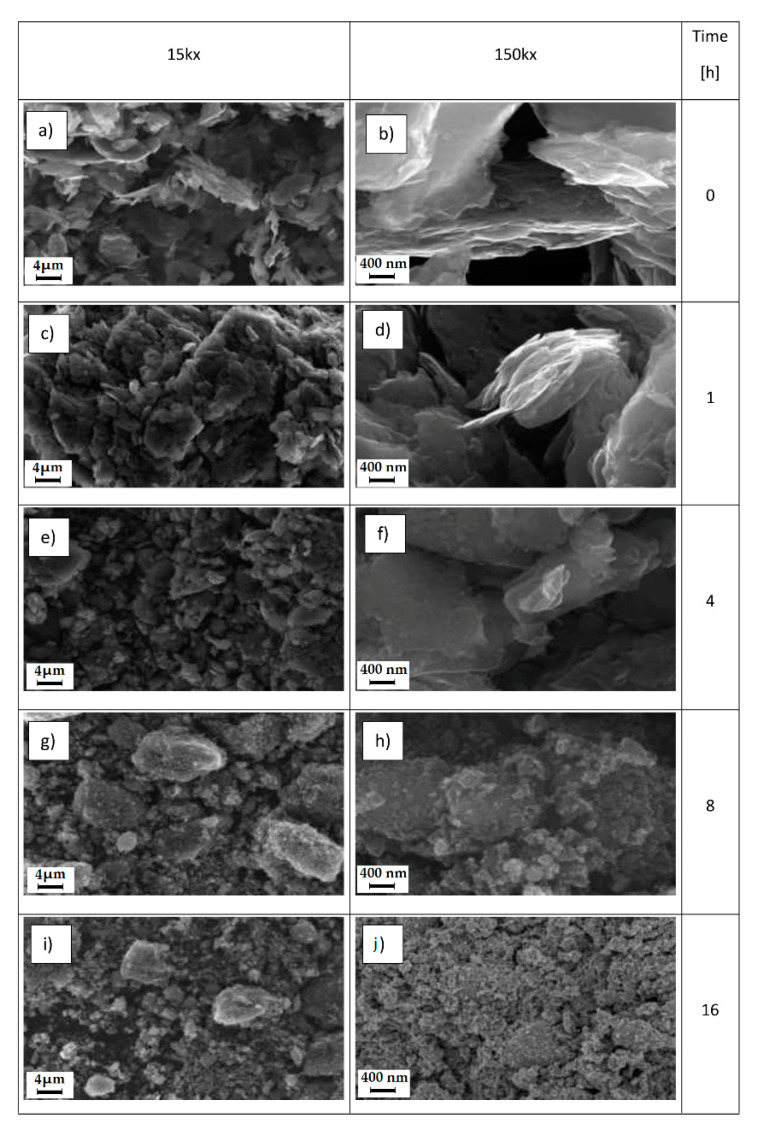
SEM images of graphite at magnifications of 15 and 150 Kx at different ball milling times: (**a**,**b**) 0 h; (**c**,**d**) 1 h; (**e**,**f**) 4 h; (**g**,**h**) 8 h; (**i**,**l**) 16 h.

SEM morphological investigations were performed on the pure and ball-milled graphite at different times. In Figure 3a,b (sample PE-G0) and in Figure 3c,d (sample PE-G1), we can see large planes of graphite, which were nearly comparable in width or slightly less after one hour of graphite ball milling treatment. From these images, it is evident that the mechanical treatment did not show significant variations after one hour compared to the pure graphite, since the graphitic planes, wide and extended (greater than 200 nm) were always observed. A similar situation was repeated after four hours of the treatment. To highlight the significant fragmentation of graphene planes, a mechanical treatment for longer times, at least 8 h (PE-G8 sample in Figure 3g,h) or even better still after 16 h (PE-G16 sample in Figure 3i,l), was required. The fragmentation into very small particles was very evident after 16 h of mechanical treatment, as the large graphitic layers disappeared in favour of small clusters smaller than 200 nm (as discussed below).

**Figure 4:**
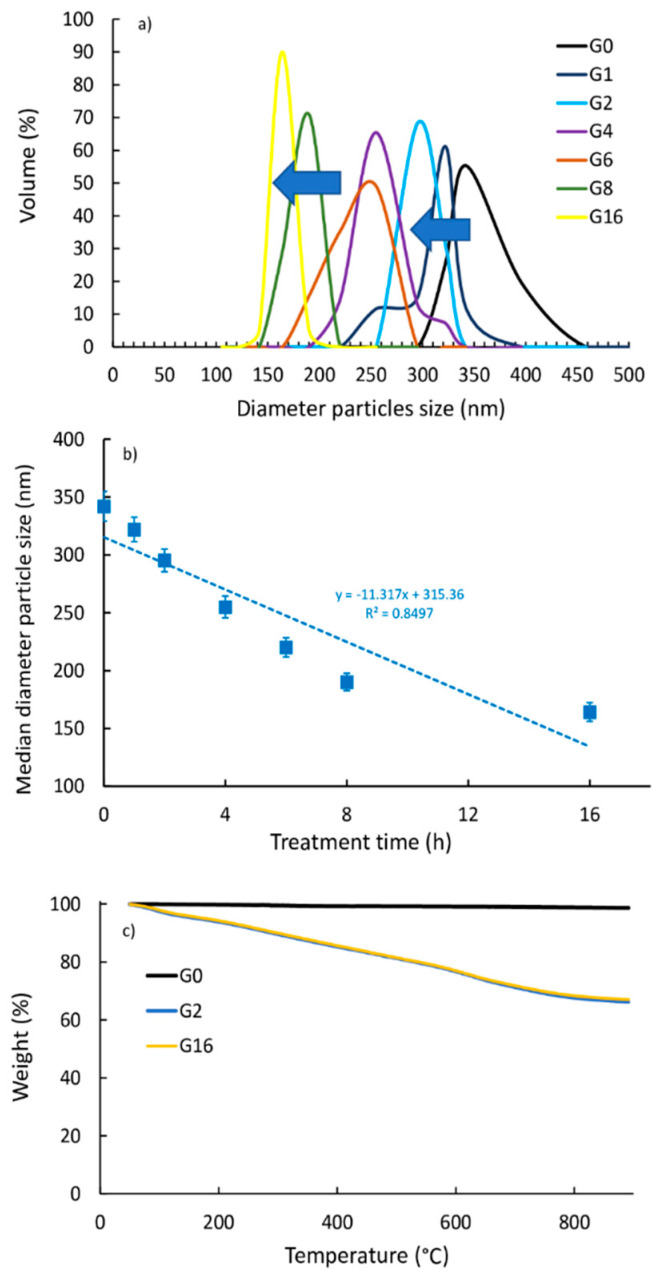
(**a**) Graphite size distribution as a function of treatment time of PE and the nanocomposites with dynamic light scattering (DLS). (**b**) Median diameter particle size vs. treatment time. (**c**) TGA analysis of PE, PE-G2, and PE-G16.

To verify the correlation between the mechanical, calorimetric, and wear properties of PE and PE–BMG composites with the effective size of the filler particles, a DLS analysis was conducted. It allows measuring the average particle size of graphite as the mechanical treatment time increased. The pure sample had an average particle size of approximately 340 nm. Then, the size decreased progressively to about 322, 295, 255, 250, 220, and 190 nm after 1, 2, 4, 6, 8, and 16h, respectively, (see Figure 4a). The DLS analysis showed a drastic reduction in size after 8 h of treatment (−40%) compared to that in the pure sample, but only a further small variation after 16 h (−51%). As shown in Figure 4b, the higher the mechanical treatment time, the lower the average diameter of the particle size. The DLS results are numerically in agreement with what was observed through the SEM analysis. The morphological analysis with SEM has in fact highlighted a drastic reduction of the graphite particles thanks to the mechanical treatment, both after 8 h and even more after 16.

Finally, the TGA analysis (Figure 4c) showed an increase in weight loss for the ball-milled graphite (ca. 35 wt %) with respect to the untreated one (ca. 2 wt %), suggesting that an oxidation reaction occurred during the ball milling treatment. It can be seen that the degree of oxidation was independent of the ball milling time and the treatment for a longer time (16 h) did not improve the oxidation with respect to the lower-one treatment (2 h). Therefore, the quiet period of 15 min that we used among the one-hour ball milling treatments of graphite powder described in Section 2 (Materials and Methods) was useful to avoid unwarranted progressive graphite’s oxidation with the increase of milling time.

**Figure 5:**
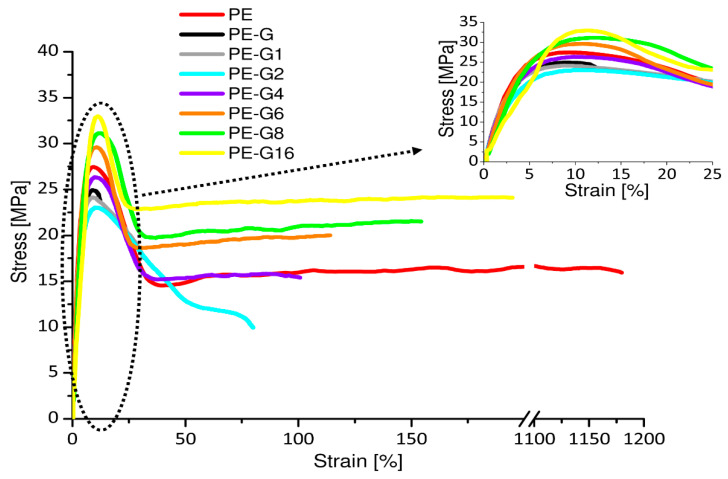
Stress–strain curves of PE and its nanocomposites.

The stress–strain graphs of the pure PE and all the mixtures (PE-G0, PE-G1, PE-G2, PE-G4, PE-G8, and PE-G16) are shown in Figure 5. The PE-G0 sample (PE mixed with pure graphite, not mechanically treated) showed a different stress–strain curve than the pure PE. In fact, an increase in the slope of the initial section and an increase in stiffness were observed (Young’s elastic modulus increased from about 500 to about 900 MPa), as visible in the magnified graph in the frame of Figure 5. The mechanical strength, both at yield and at break, decreased from approximately 28 to approximately 25 MPa and from approximately 16 to approximately 12 MPa, respectively. Strain at break decreased considerably from approximately 1200% to approximately 22% with a corresponding collapse of the work at break from approximately 24 to approximately 7 J.

The results indicated that the presence of graphite added to HDPE, even with a small quantity (0.3 wt %), caused a significant alteration of the polymeric structural organization. It acted as an inclusion, making the material very fragile. This involved the marked worsening of all the mechanical properties of the PE, which became brittle from a ductile material. The mechanical behavior of the nanocomposite mixture changed considerably, if the HDPE was mixed with the same weight of previously ball-milled graphite. In fact, mechanical stress reduced its size, and it managed to distribute itself in a homogeneous and uniform manner, intercalating itself within the macromolecular structure. The experimental evidence of what has been described revealed the reduction of modulus to approximately 630–640 MPa and the rise of the other mechanical parameters, especially after long times (8–16 h) of mechanical treatment.

Some parameters of the nanocomposite mixture became even higher than those of the pure PE. For example, in the PE-G16 sample, the mechanical strength at yield increased to a value of approximately 35 MPa, the stress at break increased up to a value of approximately 21 MPa, the maximum load reached approximately 77 N. The work at break was approximately 20 J close to that of the pure PE, and the deformation at break (i.e., approximately 200%) decreased by an order of magnitude.

As the graphite mechanical treatment time increased, there were increases in the yield stress and in the elongation at break. Therefore, the addition of graphite ball-milled for all the time periods was beneficial for increasing the mechanical properties of the pure HDPE.

**Figure 6:**
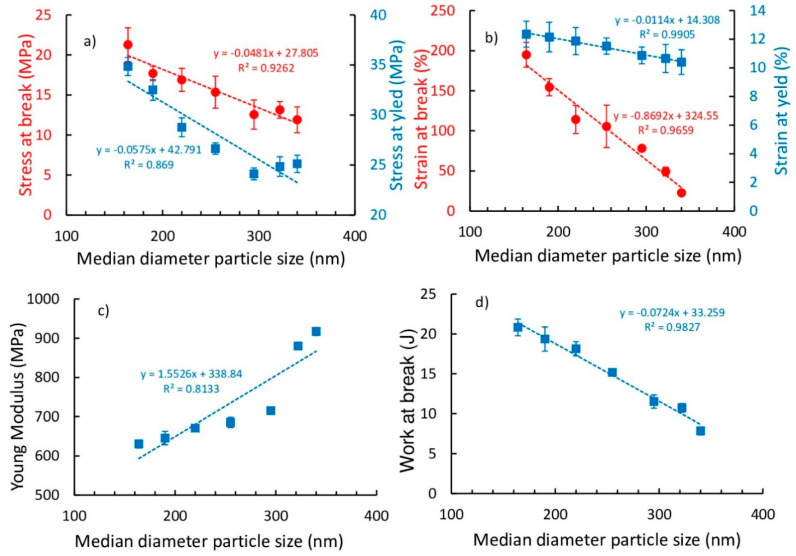
Tensile mechanical parameters of the pure HDPE and its composites with graphite treated at different times: (**a**) stresses at yield and break; (**b**) strains at yield and break; (**c**) Young’s modulus; and (**d**) work at break.

The mechanical parameters vs. the median diameter particle size is shown in Figure 6. We can observe the decreases of the stress at break and at yield (Figure 6a), the decreases in strain at break and at yield (Figure 6b), the increase in Young modulus (Figure 6c), and the decrease in work at break (Figure 6d), with the increasing of the mean diameter particle size.

Observing the values detailed in Table 2 we can conclude that:

- the increase in modulus of the PE-G16 sample (630 MPa) with respect to that of the neat PE (512 MPa) was 23%;
- the increase in strength at break of PE-G16 sample (21 MPa) with respect to thato the neat PE (16 MPa) was 33%;
- the strain at break of PE-G16 sample (−194%) respect to that of the neat PE (1177%) was decreased by about one magnitude order;
- the decrease in work at break of PE-G16 sample (20.82 J) with respect to that of the neat PE (23.84 J) was 12%.

If we compared the literature results in similar HDPE materials reinforced with the same graphite amount (0.3 wt %), we can observe that: the results in [20] showed a greater improvement in stiffness (55%), a significant decrease in deformability (greater than two magnitude orders), and a similar improvement in break strength (40%), while the results in [25] showed a greater improvement in stiffness (66%, from 600 to 1000 MPa) and a decrease in impact strength (−22%, from 450 to 350 J/m), compared to the results in the present paper. Thus, our results reflected a better filler interaction within the polymeric matrix compared to the other similar materials, since the brittleness of our nanocomposites was lower. Finally, Figure 6 highlights that there was a small difference in the mechanical behaviors of PE-G8 and PE-G16 samples.

**Figure 7:**
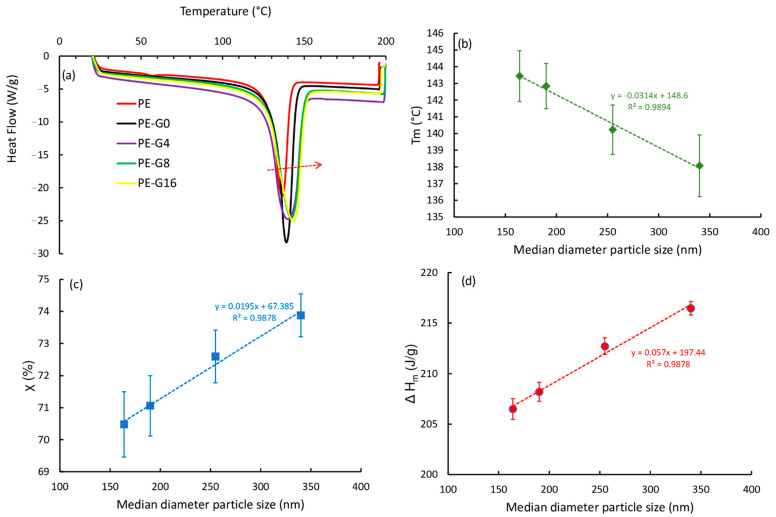
Differential scanning calorimetry (DSC) analysis of the pure HDPE and its composites with graphite ball milled at different times: (**a**) calorimetric curves; (**b**) melting temperature vs. median particle diameter; (**c**) crystalline degree vs. median particle diameter; (**d**) melting enthalpy vs. median particle diameter.

Figure 7a shows the DSC curves, while the relationships of the melting temperature and that of the degree of crystallinity with the median particle diameter are shown in Figure 7b,c, respectively. How the melting peaks of nanocomposites shifted toward right to higher vales were observed (as indicated by the row in Figure 7a), with respect to for the pure PE. Hence, the melting temperature decreased with increasing mean diameter particle size (Figure 7b), while the degree of crystallinity and the melting enthalpy increased (Figure 7c,d).

Compared to in the pure PE, increases in all parameters were identified for all the composites, as shown by the details of the values shown in Table 3. The *T_m_* value increased by 4.6%, and ∆*H* and X both increased by 11%.

These results are partially in agreement with what was reported in [20], which considered as a reference a value of ∆*H* = 288 (J/g) and had an increase of ∆*H* of 5% (in a mixture of HDPE with 60 wt % graphite EG for electrical conductivity measurements and an average size of 40 μm). Instead, the melting temperature of the same composition was almost unchanged compared to for the pure HDPE. The calorimetric data, therefore, indicated a general increase in the thermal properties of the nanocomposite, which grew as the median particle size decreased, suggesting that this improved the dispersion of the filler within the polymer matrix and therefore managed the better interaction between the filler and the matrix due to large surface area.

According to mechanical results, the DSC data in Table 3 highlighted that there was a small difference in the thermal behaviors of PE-G8 and PE-G16 samples.

**Figure 8:**
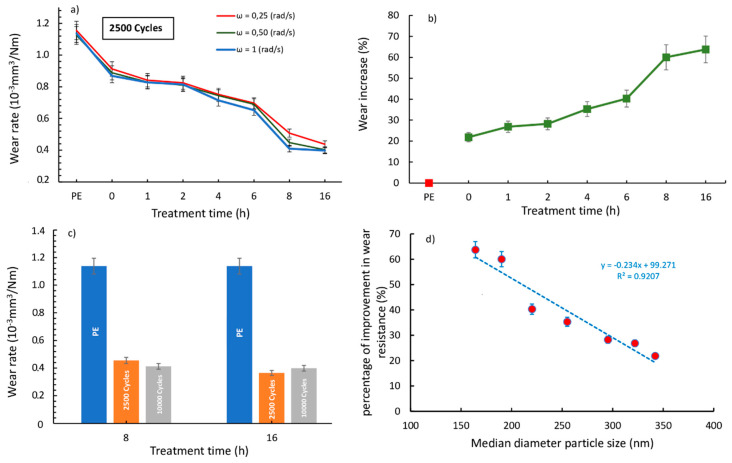
(**a**) Specific wear rates of PE and PE-G samples at different treatment times and angular speeds. (**b**) Percentage of improvement in wear resistance vs. the treatment time. (**c**) Wear rates at 2500–10.000 cycles for PE, PE-G8, and PE-G16 samples. (**d**) Percentage of improvement in wear resistance vs. the median diameter particle size.

The specific wear rate decreased compared to that of the pure PE (during 2500 cycles), as the ball milling time increased (regardless of the used speed ω; Figure 8a). The value changed from approximately 1.1 × 10^−3^ to approximately 0.4 × 10^−3^ mm^3^/Nm, with a maximum improvement of 65% in wear resistance (Figure 8b).

The wear rate decrease agreed with the torque behavior decrease observed in Figure 1. We highlighted that the presence of ball-milled graphite decreased the torque value and hence the friction of the HDPE inside the mixing chamber. The more the graphite, the longer the ball milling time. The wear rate, likewise, decreased, as the ball milling time increased. Further expanding the wear action, only two samples (PE-G8 and PE-G16) showed the best wear resistance, and then extending from 2500 to 10,000 cycles, no significant change in the wear rate value was observed (Figure 8c). This confirmed that these samples managed to have an appreciable wear resistance even after longer wear stress times.

Therefore, the mechanical action of the flaking of the graphitic planes managed to break up the graphite, which was smaller in size and better distributed within the polymeric structure, managing to give a good intercalation. In this way, the graphitic layers can act as a lubricant that improved the wear resistance of HDPE. More in-depth observations of the degree of intercalation of graphite within the polymeric matrix and its degree of exfoliation will be the subject of a future study.

Finally, the effect of the median diameter particle size on the percentage of improvement in wear resistance is plotted in Figure 8d; here, we can see that the highest wear resistance was obtained with the lowest mean diameter of the graphitic particles.

As seen through the mechanical, thermal, and wear results described above, it is evident that the eight-hour treatment could be considered enough to obtain a good reduction in particle size (19 μm). In fact, there was a good mechanical performance thanks to a sufficient distribution within the polymeric matrix and a positive interaction between the filler and the matrix. The further mechanical treatment (up to 16 h) still produced a reduction effect compared to the eight-hour treatment (16 μm), but the effect was smaller than the expected. The use of mechanical milling for a long time, such as 16 h, resulted in high mechanical forces concentrated upon the graphite layers. We should avoid the oxidation of graphite during the ball milling treatment that could occur after a long-time mechanical stress. This aspect suggests again that the treatment time of eight hours can be considered as optimal, in agreement with the mechanical tensile results.

## 4. Conclusions

In this paper, we studied PE–G nanocomposites, made with HDPE and ball-milled graphite (G). Graphite was both pure and mechanically treated in a ball mill for different times (in the range 0–16 h). Mechanical treatment was performed to reduce the size of graphite layers, which had a significant lubricating action. The aim of this work was, therefore, to verify the change in the physical–mechanical characteristics of PE-G nanocomposites, with particular attention to the variations in wear resistance. Graphite before and after the mechanical treatment has been studied by means of SEM morphological observation and the DLS analysis.

The nanocomposites were characterized with numerous investigations: during the mixing of HDPE and G, the torque was checked. Then, the static tensile, wear, and calorimetric analyses of both the pure PE and PE-G nanocomposites have been performed.

Experimental results showed that the mechanical treatment can reduce the size of the graphite particles from about 340 to 160 and 190 nm, after 8 or 16 h of mechanical ball milling treatment, respectively. The improvement in ball milling time progressively reduced the torque of the mixtures compared to in the pure PE to an ever-greater extent, when the ball milling time of the graphite was increased. This suggested an interaction between the small particles of ball-milled graphite and the polymer matrix.

From a mechanical point of view, we have verified improvements in stiffness and yield strength. The nanocomposites have also shown a progressive growth in wear resistance. In particular, the maximum improvement in wear resistance was 65%. Thermal resistance improved as well, although in little amount.

A treatment time of eight hours in ball grinding was sufficient to give an appreciable upgrade and to avoid the possible oxidative phenomena of graphite that could occur after excessive mechanical stress. The wear behavior of HDPE with ball-milled graphite has not yet been thoroughly investigated and could be important because HDPE is considered a “carrier polymer” for various low-friction applications, from biomedical use to civil engineering.

In a future work, we will focus on the optimization of the nanocomposite: the amount of mechanical treatment time (ball milling) will be fixed at eight hours, and the percentage by weight of graphite will be changed, thus testing quantities lower but also higher than 0.3%. In addition, HDPE loaded with an appropriate amount of G will be mixed with UHMWPE to verify the effect of this “carrier polymer” in the antiwear properties of PE for biomedical use or in civil engineering.

## Figures and Tables

**Table 1:** Material’s code and composition.

Code	Material’s Composition		
	High-Density Polyethylene (HDPE)	Graphite	Treatment Time (h)
Polyethylene (PE)	x	-	0
PE-G0	x	x	0
PE-G1	x	x	1
PE-G2	x	x	2
PE-G4	x	x	4
PE-G6	x	x	6
PE-G8	x	x	8
PE-G16	x	x	16

**Table 2:** Mechanical parameters obtained by the tensile test.

Code	E (MPa)	σ_y_ (MPa)	ε_y_ (%)	σ_b_ (MPa)	ε_max_ (%)	W_b_ (J)	Load (N)
PE	512.98 ± 5.36	27.47 ± 0.56	13.80 ± 1.08	16.05 ± 1.87	1177.33 ± 176.3	23.84 ± 1.29	69.62 ± 4.92
PE-G0	917.12 ± 10.22	25.12 ± 0.86	10.40 ± 0.82	11.90 ± 1.62	22.74 ± 2.14	7.85 ± 0.50	67.80 ± 1.25
PE-G1	880.25 ± 8.56	24.85 ± 0.98	10.65 ± 0.71	13.14 ± 1.05	49.25 ± 5.40	10.71 ± 0.55	71.35 ± 1.57
PE-G2	715.26 ± 9.50	24.12 ± 0.58	10.87 ± 0.55	12.56 ± 1.82	78.24 ± 3.80	11.52 ± 0.84	74.79 ± 2.57
PE-G4	685.36 ± 1.74	26.65 ± 0.57	11.52 ± 0.66	15.34 ± 2.00	105.62 ± 26.40	15.17 ± 0.16	75.67 ± 2.13
PE-G6	670.64 ± 8.22	28.77 ± 0.95	11.87 ± 0.70	16.89 ± 1.43	114.25 ± 17.49	18.14 ± 0.89	76.65 ± 3.25
PE-G8	645.62 ± 17.15	32.51 ± 1.04	12.15 ± 0.81	17.69 ± 0.91	154.47 ± 10.76	19.36 ± 1.52	76.95 ± 1.88
PE-G16	630.55 ± 10.08	34.85 ± 0.91	12.35 ± 1.23	21.29 ± 2.10	194.82 ± 15.51	20.82 ± 1.05	77.51 ± 0.85

**Table 3:** DSC results.

Code	*T_m_* (°C)	Δ*H_m_* (J/g)	Χc (%)
PE	137.16 ± 1.23	185.58 ± 4.58	63.33 ± 0.88
PE-G0	138.07 ± 1.85	216.46 ± 3.26	73.87 ± 0.67
PE-G4	140.23 ± 1.48	212.7 ± 3.91	72.59 ± 0.82
PE-G8	142.84 ± 1.36	208.2 ± 3.72	71.05 ± 0.94
PE-G16	143.44 ± 1.52	206.5 ± 3.40	70.47 ± 1.02

## Data Availability

The data presented in this study are available on request from the corresponding author.
